# Water-Use Characteristics and Physiological Response of Moso Bamboo to Flash Droughts

**DOI:** 10.3390/ijerph16122174

**Published:** 2019-06-19

**Authors:** Minxia Zhang, Shulin Chen, Hong Jiang, Yong Lin, Jinmeng Zhang, Xinzhang Song, Guomo Zhou

**Affiliations:** 1 International Institutes for Earth system Science, Nanjing University, Nanjing 210023, China; 18357173715@163.com (M.Z.); beike0922@163.com (J.Z.); 2 College of Economics and Management, Nanjing Forestry University, Nanjing 210023, China; chenshulin0923@163.com; 3 Jiangsu Provincial Key Laboratory of Geographic Information Science and Technology, Nanjing University, Nanjing 210023, China; 4 College of Forestry, Jiangxi Agricultural University, Nanchang 330000, China; 15971717613@163.com; 5 State Key Laboratory of Subtropical Silviculture, Zhejiang A&F University, Lin’an 311300, China; xzsong@126.com.com (X.S.); zhougm@zafu.edu.cn (G.Z.)

**Keywords:** flash droughts, Moso bamboo forest, evapotranspiration, water budget

## Abstract

Frequent flash droughts can rapidly lead to water shortage, which affects the stability of ecosystems. This study determines the water-use characteristics and physiological mechanisms underlying Moso bamboo response to flash-drought events, and estimates changes to water budgets caused by extreme drought. We analyzed the variability in forest canopy transpiration versus precipitation from 2011–2013. Evapotranspiration reached 730 mm during flash drought years. When the vapor pressure deficit > 2 kPa and evapotranspiration > 4.27 mm·day^−1^, evapotranspiration was mainly controlled through stomatal opening and closing to reduce water loss. However, water exchange mainly occurred in the upper 0–50 cm of the soil. When soil volumetric water content of 50 cm was lower than 0.17 m^3^·m^−3^, physiological dehydration occurred in Moso bamboo to reduce transpiration by defoliation, which leads to water-use efficiency decrease. When mean stand density was <3500 trees·ha^−1^, the bamboo forest can safely survive the flash drought. Therefore, we recommend thinning Moso bamboo as a management strategy to reduce transpiration in response to future extreme drought events. Additionally, the response function of soil volumetric water content should be used to better simulate evapotranspiration, especially when soil water is limited.

## 1. Introduction

In recent years, changes in global climate have increased the frequency of extreme weather events, including frequent summers with rapidly increasing temperatures and decreasing precipitation, which seriously threaten the health and stability of ecosystems [1]. This coupling of high temperatures and low precipitation can lead to flash droughts, which involve abnormally high evapotranspiration (ET) and low soil water moisture [2,3,4]. This process significantly reduces the net primary productivity in terrestrial ecosystems [5,6], affects plant functions, and alters forest water yields and hydrological budgets [7,8,9]. However, the frequency of flash-drought events will increase over the next few decades in both tropical and subtropical regions [2,10]. This will aggravate already limited water resources in terrestrial ecosystems [11], and may alter the distribution of plants [12]. Evapotranspiration is an important pathway in atmospheric water recycling. It controls water loss from the land surface [13] and is an important component in estimating potential productivity of forest ecosystem and budget of water resources [14]. Evapotranspiration also plays an important role in the process of water–carbon recycling and energy exchange in terrestrial ecosystems [15]. Therefore, a detailed understanding of the water consumption and physiological adjustment mechanism underlying forest response to flash drought is essential to accurately assess the role of forest ecosystems in the water cycle under the rapidly changing global climate.

Moso bamboo (*Phyllostachys edulis*) is widely distributed in the tropical and subtropical regions of East and Southeast Asia [16]. It is widely planted in China, covering an area of about 4.43 Mha, which comprises 70% of the total bamboo forest area in China and 80% of the Moso bamboo forest area globally [6]. Moso bamboo is highly valued both ecologically and economically. The annual C uptake rate of a Moso bamboo stand was reported as 5.1 t·ha^−1^ [17]. Therefore, Moso bamboo is of great significance for C absorption and mitigation of climate change [18]. Furthermore, bamboo shoots are delicious and are widely cultivated in southern China [16]. The area of bamboo forests has increased rapidly and the total area in Zhejiang Province is 60,000 ha, which is the site of the present study [19]. As the fastest growing plant in the world [20], bamboo uses a large amount of water [18]. Therefore, Moso bamboo will be extremely sensitive and respond rapidly to flash droughts. Flash-drought events occur 16–24 times every 10 years in southern China. During the summer of 2013, a flash-drought event affected 13 provinces in southern China, including Zhejiang province [21]. In recent years, study of flash-drought events has become the focus of research globally [1,2]. However, most previous studies on Moso bamboo focused on the C cycle [6,22], soil respiration, and C storage [23] under drought conditions of Moso bamboo. However, few studies have reported on the water-use characteristics and physiological adjustment mechanisms of Moso bamboo under flash drought conditions [24]. In the present study, we investigated the water-use characteristics and physiological adjustment mechanism of Moso bamboo response to flash droughts. Our findings provide a reference for developing bamboo forest management strategies, planning regional hydrological budgets, and modelling ET in regions affected by flash droughts.

## 2. Materials and Methods

### 2.1. Study Site

**Figure 1:**
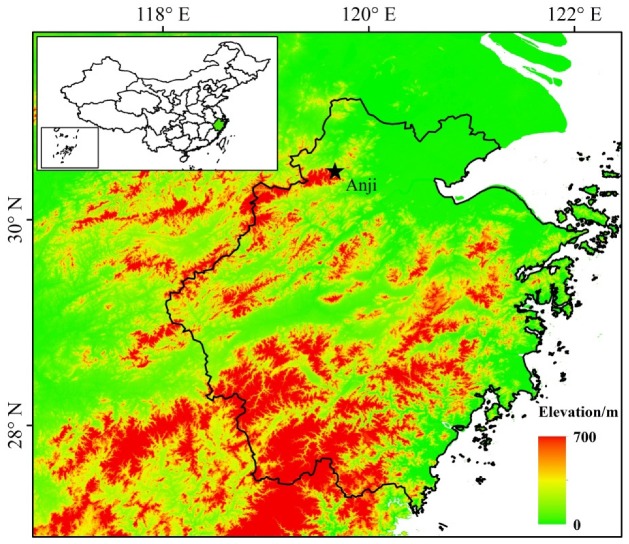
Location of the study site in Anji County, Zhejiang Province, China.

The study site was in Shanchuan town, Anji County, Zhejiang Province, China (30°28’34.5” N, 119°40’25.7” E, elevation 380 m, Figure 1). This region is characterized by a subtropical monsoon climate with four distinct seasons [25]. The mean annual temperature of the site is 16.6 °C. The mean annual precipitation is 761–1780 mm. Flash droughts often occur from mid-July to August [6]. A flash-drought event is defined as when the monthly rainfall is less than 100 mm [26]. Accordingly, the dry season in the year 2013 lasted from 10 July to 17 August (during the days the average temperature was 38.6 °C and the total precipitation was 29.3 mm). Moso bamboo covers an area of about 1693 ha (50.1% of the total area of Anji) and is the main forest type in this area with small proportions of mixed forest (1.9%) and cropland (8.6%). Mean stand density of 4500 trees·ha^−1^ [27], mean canopy height of 15.8 m, canopy coverage of 0.9, and diameter at breast height of 12–18 cm, and slope range of roughly 2.5–14.0° [22].

### 2.2. Meteorological and ET Measurements

A 38 m tall flux tower was built in the center of the study area in 2010. An eddy covariance system was mounted on the tower at a height of 38 m and used to obtain 3 years of continuous measurements from 2012–2014. The eddy covariance system included a three-dimensional sonic anemometer (CSAT3; Campbell Scientific Inc., Logan, UT, USA) to measure wind velocities and an open-path infrared gas analyzer (Li-7500; LI-COR Biosciences, Inc., Lincoln, NE, USA) to measure the densities of CO_2_ and H_2_O. The raw data were sampled at a frequency of 10 Hz and 30 min mean values were recorded using an EC100 electronics module (Campbell Scientific Inc., Logan, UT, USA) and then transmitted to a data logger (CR1000; Campbell Scientific Inc., Logan, UT, USA). The results of carbon flux, water flux, and friction wind speed were calculated on-line over a 30 min interval. For detailed information regarding the Eddy covariance system see Song et al. [6].

Micrometeorological instruments, wind speed anemometers (010C, MetOne Instruments, Grant Pass, OR, USA), and air temperature and humidity instruments (HMP45C, Vaisala, Helsinki, Finland) were mounted 1, 7, 11, 17, 23, 30, and 38 m above ground. The air temperature (Ta) and the relative humidity (RH) at 17 m was used to calculate the vapor pressure deficit (VPD). A net radiation sensor (CNR4, Kipp & Zonen, Delft, the Netherlands) was mounted at 38 m above the ground. Infrared temperature radiometers (SI-111, Apogee Inc., Logan, UT, USA) were installed at 1.5 and 5 m above the ground to record surface and canopy temperatures, respectively. Precipitation measurements were obtained from a meteorological station 150 m from the study site. Soil volumetric water content (SWC, m^3^·m^−3^) was monitored at 5, 50, and 100 cm depths using three CS-109 probes (Campbell Scientific Inc., Logan, UT, USA) and three CS-616 probes (Campbell Scientific Inc, Logan, UT, USA). Sensible heat fluxes were measured with HFP-01 flux plates (Hukseflux, Delft, the Netherlands) buried 3 and 5 cm below the ground. Latent heat flux was calculated based on the difference between the measured water vapor flux and the storage change of water vapor flux in the canopy–air space using the EC system. All 30 min data were recorded with a CR1000 data logger (Campbell Scientific Inc., Logan, UT, USA)

Leaf area index (LAI, m^2^·m^−2^), i.e., the MODIS 8 day 1 km LAI product (MOD15A2) was obtained from NASA’s website (Atmosphere Archive and Distribution System Distributed Active Archive Center, LAADS DAA, https://ladsweb.nascom.nasa.gov/data/) with an online subset output of a 1 × 1 km pixel subset centered on the flux site. The LAI time-series of the flux site was extracted using ENVI5.1 software (Exelis Visual Information Solutions, Inc., Herndon, VA, USA). Annual daily leaf area index was estimated by the linear interpolation method.

### 2.3. Data Analyses

Owing to the instrument failure, weather conditions, and other factors during the long-term and continuous measurements, some abnormal data occurred, including (1) water vapor flux data exceeding 40 g·m^−2^·s^−1^; (2) the median of the absolute deviations around the median was 2.5 times greater than the standard deviation of the five adjacent data points [25,28]. After deleting the abnormal data, the average annual valid data coverage was 61.4%. Then, we used the mean diurnal variation method (MDV) to fill the data gap. The width of the averaging window was 14 days for daytime and 7 days for nighttime for the detailed algorithm of MDV reported by Aubinet et al. [29].

Statistical Package for the Social Sciences 18.0 (SPSS Inc., Chicago, IL, USA) was further used for statistical analyses. Relationships among ET and other variables (LAI, VPD, gc) were analyzed using linear regression (i.e., coefficient of determination *R^2^* and *p*-value, respectively). The regression equation was established by multiple linear stepwise regression to select the most contribution degree of factor. The test levels of variable selection and elimination were *p* = 0.05 and *p* = 0.10, respectively.

Based on the verification of the energy balance closure of the measurement system, the following formula was used to calculate water flux (*F*_w_, g·m^−2^·s^−1^) [30]:

(1) $$F_{w}=\bar{w\prime q\prime }$$

where $w^{\prime }$ represents the turbulent quantities of vertical wind speed and q′ represents the turbulent quantities of specific humidity. Evapotranspiration (ET,mm) was calculated as the sum of the values of 30 min in each day and the final output unit was mm·day^−1^.

Crown conductance for water vapor (gc, g·m^−2^·s^−1^) was derived from the measured eddy covariance (*Ec*, g·m^−2^·s^−1^) [31]:

(2) gc=Ec/Δw

(3) Δw=VPD/Pa

where Pa is the air pressure (kPa), VPD represents the water vapor pressure deficit of the air, and Δw represents the leaf/air mole fraction difference of water vapor at canopy height.

Ecosystem-scale water-use efficiency (WUE) was defined as the ratio of gross primary productivity (GPP) to ET [32,33]:

(4) WUE=GPP/ET

where GPP is the monthly gross primary productivity (gC m^−2^·mon^−1^) and ET is the monthly evapotranspiration (mm·mon^−1^).

Canopy interception was calculated as follows [26]:

(5) $$I_{p}=P-0.94\times P-0.76$$

where $I_{p}$ represents the canopy interception rainfall and *P* is the monitored rainfall.

Potential evapotranspiration (PET, mm·day^−1^) representing the index of demand that drives water back to the atmosphere when sufficient water and energy are available [34,35], and it was calculated using an equation from the United Nations’ Food and Agriculture Organization (FAO) [36]:

(6) $$\frac{0.408\Delta \left(\mathrm{Rn}-G\right)+\gamma \frac{900}{Ta+273.15}\mu 2\left(\mathrm{es}-\mathrm{ea}\right)}{\Delta +\gamma \left(1+0.34\mu 2\right)}$$

where Δ (kPa·°C^−1^) is the slope of the saturation water vapor pressure versus the *Ta* curve, Rn (MJ·m^−2^·day^−1^) is net radiation, G (MJ·m^−2^·day^−1^) is soil heat flux, γ (kPa·°C^−1^) is the psychometric constant, *Ta* is the average air temperature (°C), es–ea (kPa) are the saturation vapor pressure and actual vapor pressure, respectively, and *u*2 is the mean wind speed (m·s^−1^) at 2 m height.

The dryness index (DI) was estimated as follows:

(7) DI=PET/P

where P is precipitation (mm·month^−1^) [28].

## 3. Results

### 3.1. Meteorological Variables

**Figure 2:**
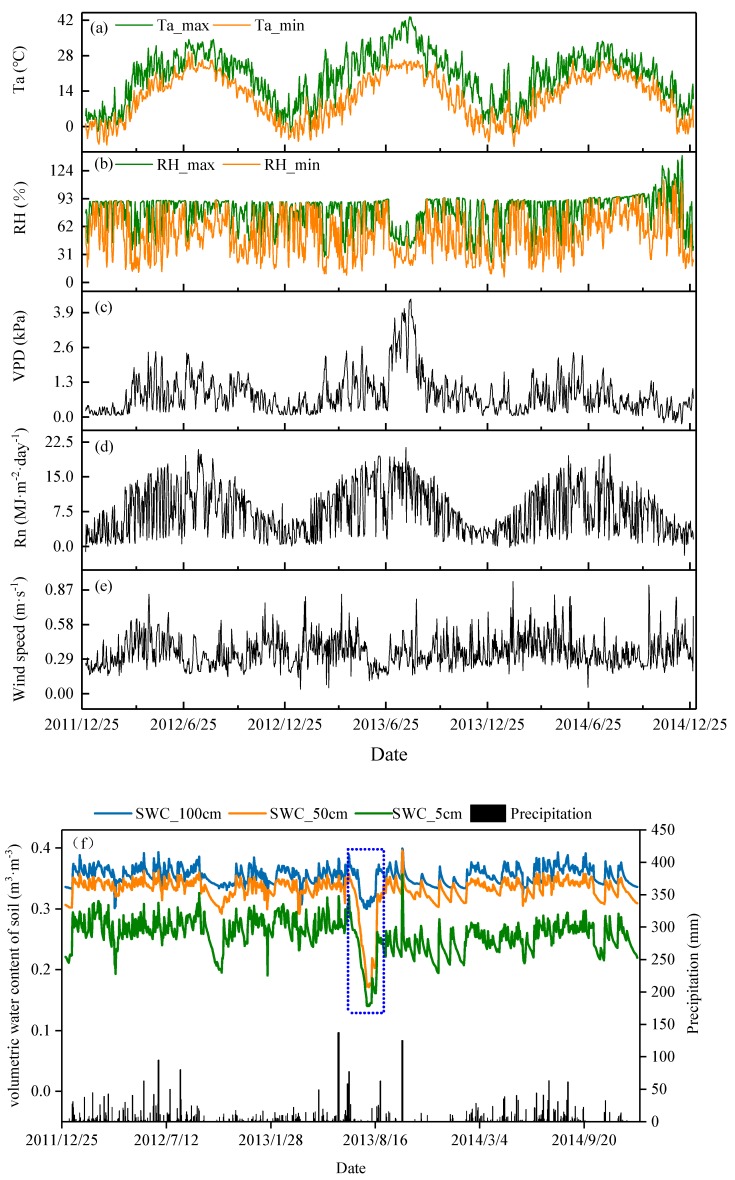
The daily dynamics of environmental variables in the Moso bamboo forest in Anji country from 2012–2014. (**a**) Air temperature: green line represents the maximum daily temperature (Ta_max) and the orange line represents the minimum daily temperature (Ta_min); (**b**) relative humidity: green line represents the maximum relative humidity (RH_max) and the orange line represents the minimum relative humidity (RH_min); (**c**) vapor pressure deficit (VPD, kPa); (**d**) net radiation (Rn, MJ·m^−2^·day^-1^); (**e**) wind speed (m·s^−1^), (**f**) precipitation (P, mm), and soil volumetric water content (SWC, m^3^·m^−3^)> SWC_5 cm, SWC_50 cm, and SWC_100 cm are the volumetric water content of soil at a depth of 5 cm, 50 cm, and 100 cm.

Daily mean temperature ranged from 11.4 °C to 22.5 °C in 2012–2014. The flash drought occurred from 10 July to 17 August in 2013, and temperatures above 40 °C lasted for 12 days, mainly occurring in early August. During the days, the average temperature was 38.6 °C and the highest temperature reached 43.1 °C. The hot weather gradually receded after August 17. Mean temperatures were 30.7 °C and 28.7 °C at the same period in 2012 and 2014, respectively (Figure 2a). Daily mean RH ranged from ~47.5–86.4% in 2012–2014. The average daily RH was 37.8% during the flash drought periods, which was lower than the RH of 76.4% and 76.3% in the same period of 2012 and 2014, respectively (Figure 2b). The daily average VPD ranged from 0.6–1.1 kPa. The maximum VPD reached 4.30 kPa in 2013, which is significantly higher than the VPD of 2.45 kPa and 2.41 kPa observed in 2012 and 2014, respectively (Figure 2c). Total Rn was 3071 MJ·m^−2^ in 2013, which was higher than the 360 MJ·m^−2^ and 537 MJ·m^−2^ observed in 2012 and 2014, respectively (Figure 2d). The wind speed was lower in the study site at an average of 0.34–0.37 m·s^−1^ (Figure 2e).

The variation in annual and monthly precipitation from 2012 to 2014 is shown in Figure 2f. Total precipitation (P) of each year was 1625 mm, 1314 mm, and 1349 mm in the study site from 2012–2014. There was an inter-annual change in precipitation, in that the annual precipitation decreased. The annual average P for 2012–2014 was 1426 mm, which was close to the long-term average value recorded at the nearest meteorological observatory (761–1780 mm). Precipitation occurred more frequently during summer than winter. There were 10 times the amount of rainfall from 10 July to 17 August, and the total precipitation was 29.3 mm during the flash drought periods in 2013, which is the lowest value recorded for 50 years. Heavy rainfall occurred on 10 August 2013 with a total precipitation of 20.7 mm. On other days, precipitation was less than 3 mm.

The dynamic of soil volumetric water content (SWC, m^3^·m^−3^) generally changed with precipitation and the difference in SWC was obvious at different soil depths (Figure 2f). The average SWC_5 cm, SWC_50 cm, and SWC_100 cm was 0.26 m^3^·m^−3^, 0.33 m^3^·m^−3^, and 0.35 m^3^·m^−3^ from 2012 to 2014, respectively. The value of SWC_5 cm decreased from 0.29 m^3^·m^−3^ to 0.14 m^3^·m^−3^, SWC_50 cm decreased from 0.35 m^3^·m^−3^ to 0.17 m^3^·m^−3^, and SWC_100 cm decreased from 0.37 m^3^·m^−3^ to 0.30 m^3^·m^−3^. Soil volumetric water content at 0–50 cm depth decreased by 0.15 m^3^·m^−3^ and 0.18 m^3^·m^−3^ from 1 July to 31 July 2013, respectively, which was greater than the value of 0.07 m^3^·m^−3^ for SWC_100 cm (Figure 2f). It can be seen that the loss of water in SWC_0–50 cm was more obvious than that in SWC_100 cm. This phenomenon was also observed in October 2012. The values of meteorological variables (Ta, RH, VPD, Rn, and P) were higher in summer than in winter and were determined by the characteristics of the typical climate in China.

### 3.2. The Monthly Dynamic Characteristics of WUE and Precipitation

**Figure 3:**
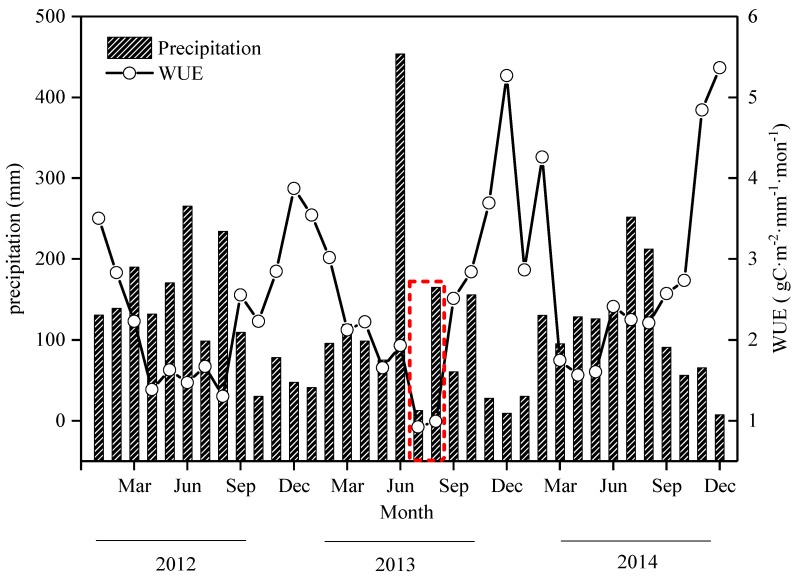
The monthly dynamics of water-use efficiency (WUE, gC·m^-2^·mm^-1^·mon^-1^) and Precipitation in the Moso bamboo forest in Anji country from 2012–2014. The red, dotted frame represents the monthly variation in WUE during the flash-drought period.

The characteristics of precipitation experienced obvious seasonal variability (Figure 3). The total maximum precipitation, generally occurring in summer, were 598.6 mm, 630.8 mm, and 610.5 mm in 2012–2013, and accounting for 36.7–48.0% of the total annual precipitation, respectively. The total minimum precipitation in winter accounted for about 11.1–19.7% of the year. However, the total precipitation was 12.6 mm in July in 2013. The WUE and precipitation of Moso bamboo forest showed an opposite trend at the seasonal scale in this study (Figure 3). The monthly averaged WUE during the period was 2.29 gC m^−2^·mm^−1^·mon^−1^, 2.56 gC·m^−2^·mm^−1^·mon^−1^, and 2.87 gC·m^−2^·mm^−1^·mon^−1^ in the study site from 2012–2014, respectively. Water-use efficiency decreased significantly with the reduction of precipitation in July and August 2013, and the values were 0.92 gC·m^−2^·mm^−1^·mon^−1^ and 0.99 gC·m^−2^·mm^−1^·mon^−1^, which were much lower than the WUE in 2012 and 2014. It can be seen that WUE during the flash drought period was significantly different from that in conventional climate in 2012 and 2014.

### 3.3. Dynamic Characteristics of ET, PET, and LAI

**Figure 4:**
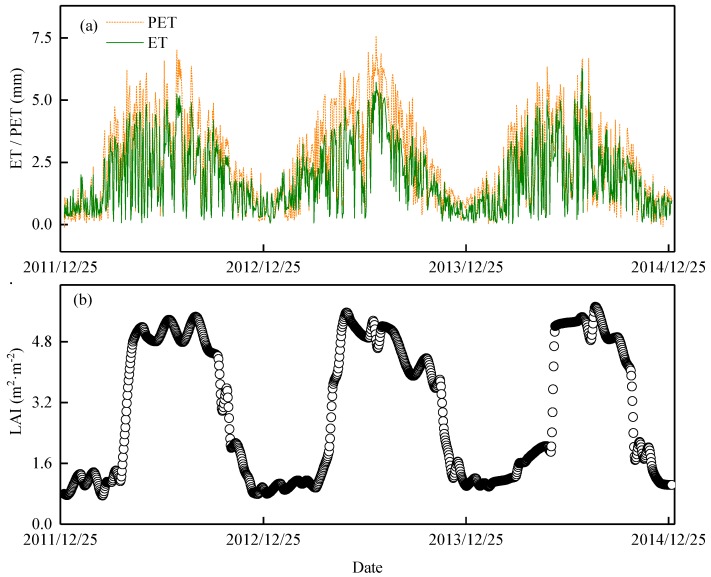
The dynamics of daily actual evapotranspiration (ET, mm), potential evapotranspiration (PET, mm) and leaf area index (LAI, m^2^·m^−2^) in the Moso bamboo forest from 2012 to 2014. (**a**) Daily ET and PET, green line represents the ET and the orange, short dashed line represents the PET, respectively; (**b**) daily LAI.

The characteristics of ET were also seasonally variable, consistent with the meteorological factors (Figure 2). The average daily ET was from 1.79–2.00 mm·day^−1^ from 2012–2014 (Figure 4a). During the flash drought periods, ET was recorded at 4.41 mm·day^−1^, initially increasing and reaching its maximum of 5.73 mm·day^−1^ on 16 July, before gradually decreasing to 1.51 mm·day^−1^ on 17 August. This peak in ET in the summer was influenced by meteorological factors and accounted for 37.7–42.9% of the total annual ET (Figure 4a). Evapotranspiration was at its lowest in the winter, accounting for about 9.5–11.5% of the annual ET. The total ET reached 139 mm in July during the flash drought periods in 2013, which was higher than the 111 mm and 94 mm observed in the same month in 2012 and 2014, respectively.

Potential evapotranspiration was estimated by Penman’s formula (Equation (6)) in 2012 to 2014 to be 865 mm, 1017 mm, and 814 mm, respectively (Figure 4a). The total PET was 188 mm, 287 mm, and 160 mm higher than the ET observed in 2012 to 2014, respectively. The monthly and seasonal characteristics of the PET were basically consistent with the ET, reaching its highest value in summer and lowest in winter. The highest observed daily PET during a flash drought period in 2013 was 7.59 mm·day^−1^.

The LAI dynamics had evident seasonal differences (Figure 4b). The average LAI was 3.02 m^2^·m^−2^ in 2012–2014. The maximum LAI was from 5.08–5.31 m^2^·m^−2^ in 2012–2014 and generally occurred in the growing season from June–August. It reached its maximum in August in 2012 and 2014 but in June in 2013. The LAI was 4.87 m^2^·m^−2^ in August 2013, which was lower than the 0.26 m^2^·m^−2^ LAI observed in July 2013. It significantly decreased by 0.31 m^2^·m^−2^ and 0.44 m^2^·m^−2^ compared with the same period in 2012 and 2014, respectively. The minimum LAI, from 0.87–1.08 m^2^·m^−2^, was recorded in winter.

## 4. Discussion

### 4.1. The Response of SWC and LAI of Moso Bamboo Forests to Flash Drought.

Soil moisture is a direct source of water for plant growth [12], and it reflects soil water availability for tree transpiration [37]. Soil moisture is jointly controlled by rainfall, ET, and runoff [38]. The influence of soil water on ET varies with rainfall amount and ET is most clearly linked to soil water when rainfall is limited [39]. Rainfall was rare and soil water sources tended to be more depleted during the flash drought periods (10 July to 17 August 2013). Soil volumetric water content at 5–50 cm depth decreased rapidly and was significantly lower than that in SWC_100 cm (Figure 2f). The root hairs of Moso bamboo are usually distributed above 40 cm soil depth [40], and there is generally less soil evaporation in a closed canopy forest [41,42]. Therefore, canopy transpiration of Moso bamboo may be the main reason for SWC_5–50 cm decreasing rapidly during the flash drought periods, which in turn, will affect the growth of Moso bamboo.

**Figure 5:**
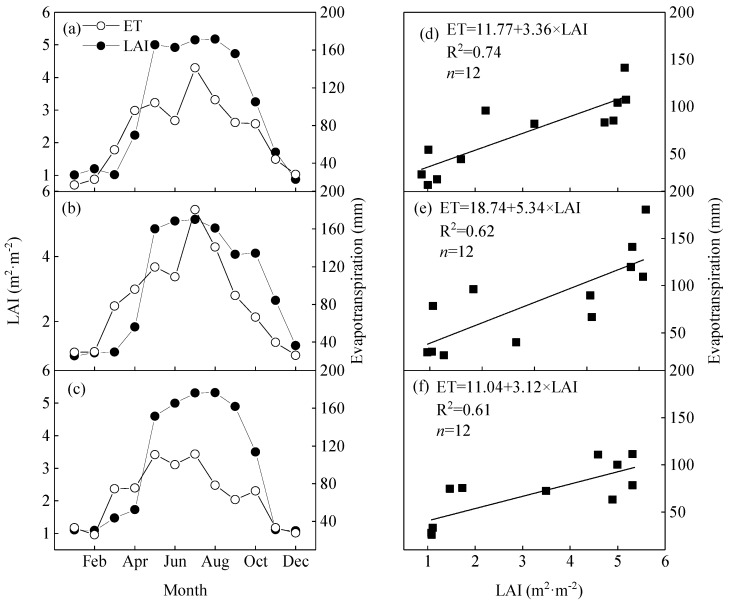
The relationship between (**a**–**c**) leaf area index (LAI) and (**d**–**f**) actual evapotranspiration (ET) from 2012–2014.

According to the MODIS LAI product, the average LAI also significantly decreased to 4.87 m^2^·m^−2^ in August 2013, which was lower by 0.26 m^2^·m^−2^ LAI observed in July 2013. It also significantly decreased by 0.31 m^2^·m^−2^ and 0.44 m^2^·m^−2^ compared with the same period in 2012 and 2014, respectively (Figure 5a–c). The maximum LAI generally occurred in the growing season, peaking in August in 2012 and 2014, but the maximum value in June 2013. The decrease in LAI was probably due to the physiological dehydration of Moso bamboo caused by insufficient SWC during this period. Qiang et al. [24] found that the aboveground biomass of Moso bamboo decreased during drought periods, and other previous studies also reported defoliation of plants during drought periods [43]. The decrease in SWC was accompanied by a decrease in ET, especially from 16 July to 16 August. The daily ET decreased from 5.73 mm·day^−1^ to 1.51 mm·day^−1^ during the flash drought periods (Figure 4a). Linear regression analysis was conducted between ET and LAI. The results showed a significant linear correlation between ET and LAI (*R^2^* > 0.58, *p* < 0.01, Figure 5d–f). The leaf surface is the site of transpiration and photosynthesis, and its area generally determines the capacity of the canopy to capture energy and the physiological potential of water vapor exchange [37]. Therefore, it can be inferred that the rapid decrease in daily ET in August was related to Moso bamboo defoliation [37,44].

Further analysis revealed that the SWC_5 cm and SWC_50 cm decreased by 0.14 m^3^·m^−3^ and 0.18 m^3^·m^−3^, respectively, in August, which were at the lowest level in summer and remained stable until the rainfall on 18 August (Figure 2f). Therefore, SWC_50 cm < 0.17 m^3^·m^−3^ may be the inducing factors of bamboo deciduous. Soil water content determines forest water use [12]. Owing to the storage capacity of the soil being limited to two weeks [45], the bamboo needed water from antecedent soil water stores to maintain high levels of transpiration [45,46]. During long-term extreme drought conditions, soil water is depleted and there is no replenishment from rainfall resulting in physiological dehydration of plants. Furthermore, transpiration loss can be reduced by defoliation [47]. Previous studies have reported that the mean sap flow decreased steadily when soil moisture had decreased [48]. Pataki et al. [49] reported an observed decrease in maximum sap flow for *Pinus contorta*, *Abies lasiocarpa*, *Populus tremuloides*, and *Pinus flexilis* when soil moisture decreased from 0.35 to 0.24 m^3^· m^−3^ at 0–45 cm. Gartner et al. [48] found that the mean sap flow of trees decreased from 0.567 kg cm^−1^ to 0.258 kg cm^−1^ in response to drought conditions. Canopy transpiration also decreased at the same time [50]. For example, Scots pine (*Pinus sylvestris*.) and Norway spruce (*Picea abies*.) transpiration was reduced by 40% and 67% during the dry summer [51]. The findings from the present study are supported by the results from previous studies.

The WUE and precipitation of Moso bamboo forest showed an opposite trend at the seasonal scale in this study. The relationship between WUE and precipitation observed in our study was consistent with previous results based on the site observed [52]. Water-use efficiency significantly decreased to 0.92 gC·m^−2^·mm^−1^·mon^−1^ and 0.99 gC·m^−2^·mm^−1^·mon^−1^ with the reduction of precipitation in July and August 2013. Reichstein et al. [53] found that WUE of plants was negatively correlated with SWC and decreased significantly with the reduction of SWC during the flash drought period. Lu et al. [54] also found that the WUE of plants showed a decreased tendency under severe drought conditions using observation and remote sensing data. The results from this study are consistent with the findings from previous studies. The reason may be that stomata is much more sensitive to drought when Moso bamboo is under the condition of mild and moderate water stress, which will be further discussed below. Physiological dehydration occurred in Moso bamboo to reduce ET by defoliation, which results in a significant decrease in WUE.

Water vapor exchange through transpiration and CO_2_ absorption through photosynthesis are carried out in leaf stomata. Therefore, the transpiration rate directly affects plant photosynthesis and then affects the net primary productivity of an ecosystem [37]. Song et al. [6] reported that an extreme drought that occurred in July and August 2013 significantly reduced the net ecosystem productivity of a Moso bamboo forest by 60–78%. Xu et al. [22] reported that a flash drought resulted in both net and gross primary productivity of a Moso bamboo forest decreasing by 12% and 48%, respectively. Noormets et al. [21] observed that a mosaic of oak (*Quercus* spp.) forests stands accumulated 40% less carbon in a year under flash drought conditions. Leaf area index affects the WUE (WUE = GPP/ET) of an ecosystem by controlling GPP and ET to response to flash-drought events. The consistent response of ET, WUE, GPP, and net ecosystem exchange to extreme drought also indicates the coupling of water and carbon in ecosystems.

### 4.2. Response of Evapotranspiration and Canopy Stomatal Conductance to Drought

Canopy conductance (gc) and VPD are the main variables limiting transpiration [14]. Vapor pressure deficit reflects the level of atmospheric evaporative demand for water vapor [55]. The gc controls the transpiration by regulating the exchange of water and vapor between the atmosphere and soil in response to extreme drought conditions [41]. Stomatal behavior is constrained by ambient air and soil conditions [14]. When soil water moisture decreases, gc is usually more sensitive to VPD [55]. We analyzed the relationship between ET, gc, and VPD to investigate how the water exchange between leaves and the atmosphere is controlled by stomata during their response to flash drought conditions when soil water is limited.

**Figure 6:**
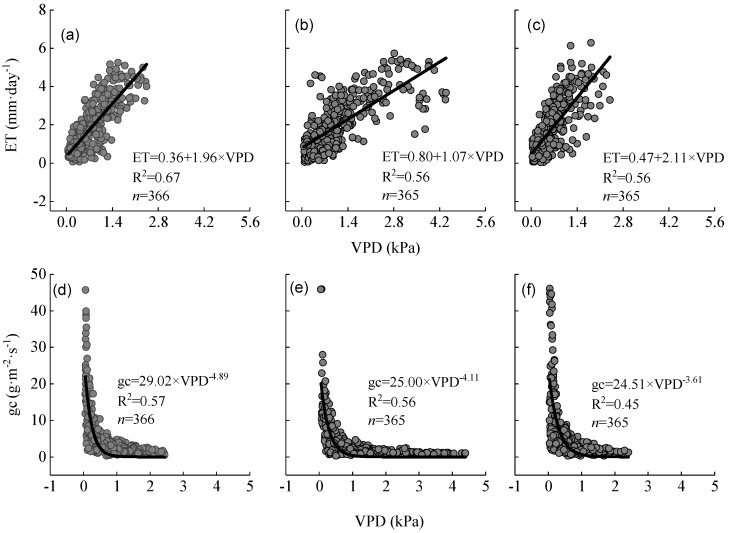
(**a**–**c**) Relationships between the vapor pressure deficit (VPD) and actual evapotranspiration (ET) from 2012–2014, respectively; solid lines are regression lines. (**d**–**f**) the response of canopy conductance (gc) to VPD.

Linear regression analysis was conducted between ET and VPD. The results showed a strong linear correlation between ET and VPD (*R^2^* > 0.50, *p* < 0.01; Figure 6a–c). Evapotranspiration increased with increasing VPD up to 2.0 kPa, and the value at which ET increased slowly or did not further increase was probably determined by the stomata. Canopy conductance decreased at an exponential rate with increasing VPD (Figure 6d–f). When VPD > 2 kPa, gc tended to be 0. This suggests that the response of ET to VPD is consistent with the response of gc to VPD.

It can be inferred that this threshold (VPD = 2 kPa) may be a reference value useful for judging the flash drought affecting the Moso bamboo forest in this study site. When the transpiration rate was higher and water resources were limited, the stomatal regulation mechanism of Moso bamboo was triggered to decrease transpiration by adjusting stomatal conductance through changing the width of the stomatal opening in response to the changing atmospheric and soil water conditions [56].

The sensitivity of gc to VPD reflects the drought resistance and water-use strategies of plants and determines their growth. When VPD is higher, the carbon assimilation rate and photosynthetic yield is also higher [57]. The threshold of VPD response to ET observed in our study was consistent with results reported by Xu et al. [22], Komatsu et al. [58], and Ichihash et al. [59]. However, it was higher than that reported from temperate coniferous forests, hardwood forests, and poplar forests (VPD < 0.6 kPa) [60]. This suggests that Moso bamboo is more drought resistant than these other types of forest.

To compare the effects of meteorological and biological factors on evapotranspiration, and to determine the contribution rate of each factor to Moso bamboo forest evapotranspiration in the study area, the relationships between VPD, LAI, gc, and ET were analyzed by multiple stepwise regression analysis. The optimal ET model was obtained as follows: ET = 0.325 VPD−0.024 gc + 0.028 LAI + 0.271 (*R^2^* = 0.56, *p* < 0.01). Evapotranspiration is a function of VPD, gc, and LAI. Partial regression coefficients showed that VPD, gc, and LAI explained 32.5%, 2.4%, and 2.8% of the changes in ET, respectively. It was found that VPD had the most significant effect on evapotranspiration and for every 1 kPa increase in VPD, ET would increase by 0.33 mm per unit area of Moso bamboo forest.

### 4.3. Water Budgets and Partitioning of the Moso Bamboo Forest

**Figure 7:**
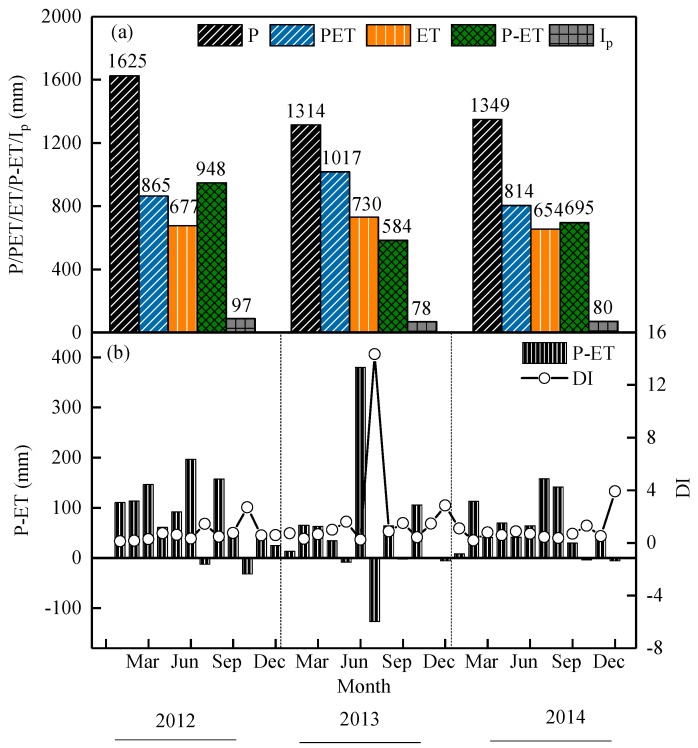
Water budgets between precipitation and evapotranspiration of Moso bamboo forest in drought periods. (**a**) Total precipitation (*P*), evapotranspiration (ET), potential ET (PET), canopy intercepted rainfall (I_p_), and precipitation-deducted ET P-ET; (**b**) monthly P-ET and dryness index (DI) through time.

The variation in precipitation quantity is significant in terms of altering the other water budget components: ET and soil moisture [61]. Maintaining a dynamic balance between precipitation and water consumption is the basis of ensuring ecosystem health and sustainable development [12]. Therefore, understanding the water-use dynamics of plants and the interactions between forests and their hydroclimate, as well as the role of forests in water partitioning, is essential for water resource and forest management [14]. To evaluate the effect of flash drought conditions on water budgets of Moso bamboo, we determined precipitation, ET, PET, I_p_, and soil water storage from 2012–2014 (Figure 7a). Total precipitation (*P*) during the period was 1625 mm, 1314 mm, and 1349 mm in the study site in 2012–2014, respectively. Annual PET was 865 mm, 1017 mm, and 814 mm in 2012–2014, respectively (Figure 7a). Evapotranspiration is controlled by relative proportion and timing of available water and available energy [62,63], and the annual ET was 677 mm, 730 mm, and 654 mm in 2012–2014, respectively (Figure 7a). To understand the water sources for forest growth, we calculated the monthly water balance between rainfall and transpiration. We hypothesized that all precipitation-deducted ET (P-ET) is stored in the soil. The soil water storage was 760 mm, 297 mm, and 533 mm from 2012 to 2014, respectively (Figure 7a). The proportion of average annual water storage was about 51.4%. Over the three years, there was a surplus of precipitation, which does not seem to cause a shortage of water in the ecosystem. However, in a few months of the year, total transpiration exceeded rainfall, and the distribution of monthly P-ET was negative. Although summer is an important season for rainfall, the huge ET caused by the flash drought can cause net loss of water in some months. Especially in the flash drought period of 2013 when P-ET was −168 mm (Figure 7b). Dryness index reached 14.34 in July, which was significantly higher than the annual average of 2.17 in 2013. It was also higher than the average of 0.73 and 0.95 in 2012 and 2014, respectively (Figure 7b). Mehan et al. [64] also reported that the decrease of precipitation and the increase of evapotranspiration will lead to the decrease of surface run-off and available water. This extra loss comes from the deeper soil layers, which leads to SWC_0–50 cm sharply decreasing during the flash drought period. The SWC_0–50 cm decreased rapidly (Figure 2f), and persistent drought conditions even caused physiological dehydration of Moso bamboo to deciduous to resistant flash drought.

The I_p_ of Moso bamboo was small (5.3–5.4%) and there was no significant difference in the three-year investigation at this study site. This was lower than the I_p_ of 11.9% observed for Moso bamboo reported by Wang and Liu [41] in Jiangxi Province in China, and the I_p_ of 9.2–12.2% reported by Shinohara et al. in Japan [65]. These inconsistencies may due to the methodological differences. According to the calculation of water budget over three years, about 50.0–66.1% of precipitation in the study area returned to the atmosphere through ET. This was basically consistent with the estimation of ET using the Eddy covariance method in 2011 (745/1543) as reported by Liu et al. [27]. Forest ET is one of the main driving factors of precipitation [12,66].

The phenomenon of physiological dehydration and defoliation appeared in Moso bamboo forests after about 30 days of extreme drought conditions. Soil volumetric water content at 50 cm depth decreased from 0.35 m^3^·m^−3^ to 0.17 m^3^·m^−3^ in the initial stage of the flash drought. Mean stand density was about 4500 trees·ha^−1^ during the period. We hypothesized that the water consumption of Moso bamboo was linearly related to the density in this study site. When mean stand density was <3500 trees·ha^−1^, the bamboo forest could safely survive extreme drought conditions. Therefore, we recommend thinning of Moso bamboo as an effective management strategy in Moso bamboo forests.

## 5. Conclusions

With decreasing soil moisture, Moso bamboo undergoes two stages of physiological regulation to reduce transpiration. First, when VPD > 2Pa and PET > 4 mm·day^−1^, the canopy transpiration of Moso bamboo may be actively regulated by stomatal closure. When SWC_5 cm was <0.17 m^3^·m^−3^, passive physiological regulation of bamboo defoliation was caused by flash drought, which led to the WUE decrease of Moso bamboo. Flash-drought events lasting for about 39 days not only affected plant growth in the short term, but also had a significant impact on the annual water budget of Moso bamboo forests. The ET of Moso bamboo was up to 730 mm in 2013, which was higher than that of 677 mm and 654 mm observed in 2012 and 2014, respectively. The soil water storage was 584 mm, significantly lower than the observed value of 948 mm and 695 mm in 2012 and 2014, respectively.

When mean stand density is <3500 trees·ha^−1^, the bamboo forest can safely survive extreme drought conditions. Therefore, we recommend thinning Moso bamboo as an effective management strategy to reduce transpiration and protect Moso bamboo from future drought events. In addition, the response function of soil volumetric water content should be incorporated into the model to better simulate ET, especially when soil water is limited.
